# Longitudinal follow‐up of sexual function after surgery for ultra‐low rectal cancers located within 5 cm of the anal verge: A multicentre collaborative study

**DOI:** 10.1111/codi.70092

**Published:** 2025-04-18

**Authors:** Yasuhiro Ishiyama, Yasumitsu Hirano, Yuichiro Tsukada, Jun Watanabe, Yosuke Fukunaga, Kazuhiro Sakamoto, Hiroki Hamamoto, Masanori Yoshimitsu, Hisanaga Horie, Nobuhisa Matsuhashi, Yoshiaki Kuriu, Shuntaro Nagai, Madoka Hamada, Shinichi Yoshioka, Shinobu Ohnuma, Tamuro Hayama, Koki Otsuka, Yusuke Inoue, Kazuki Ueda, Yuji Toiyama, Satoshi Maruyama, Shigeki Yamaguchi, Keitaro Tanaka, Takeshi Naitoh, Masahiko Watanabe, Motoko Suzuki, Toshihiro Misumi, Masaaki Ito, Ultimate Trial Group in Japan Society of Laparoscopic Colorectal Surgery

**Affiliations:** ^1^ Division of Gastroenterological Surgery Saitama Medical University International Medical Centre Saitama Japan; ^2^ Department of Colorectal Surgery National Cancer Centre Hospital East Chiba Japan; ^3^ Department of Surgery Gastroenterological Centre, Yokohama City University Medical Centre Kanagawa Japan; ^4^ Department of Colorectal Surgery Kansai Medical University Osaka Japan; ^5^ Department of Gastroenterological Surgery Cancer Institute Hospital, Japanese Foundation of Cancer Research Tokyo Japan; ^6^ Department of Coloproctological Surgery Juntendo University Faculty of Medicine Tokyo Japan; ^7^ Department of General and Gastroenterological Surgery Osaka Medical and Pharmaceutical University Osaka Japan; ^8^ Department of Surgery Hiroshima City North Medical Centre Asa Citizens Hospital Hiroshima Japan; ^9^ Department of Surgery Jichi Medical University Tochigi Japan; ^10^ Department of Gastroenterological Surgery, Pediatric Surgery Gifu University, Graduate School of Medicine Gifu Japan; ^11^ Department of Surgery Kyoto Prefectural University of Medicine Kyoto Japan; ^12^ Department of Surgery and Oncology Graduate School of Medical Sciences, Kyushu University Fukuoka Japan; ^13^ Department of Gastrointestinal Surgery Kansai Medical University Hospital Hirakata Japan; ^14^ Department of Surgery Yao Municipal Hospital Osaka Japan; ^15^ Department of Surgery Tohoku University Hospital Miyagi Japan; ^16^ Department of Surgery Teikyo University School of Medicine Tokyo Japan; ^17^ Department of Advanced Robotic and Endoscopic Surgery Fujita Health University School of Medicine Aichi Japan; ^18^ Department of Surgery Nagasaki University Graduate School of Biomedical Sciences Nagasaki Japan; ^19^ Division of Endoscopic & Colorectal Surgery Department of Surgery, Kindai University, Faculty of Medicine Osaka Japan; ^20^ Department of Gastrointestinal and Paediatric Surgery Mie University Mie Japan; ^21^ Gastroenterological Surgery Niigata Cancer Centre Hospital Niigata Japan; ^22^ Division of Colorectal Surgery Department of Surgery, Tokyo Women's Medical University Tokyo Japan; ^23^ Department of General, Breast and Digestive Surgery Otsu City Hospital Shiga Japan; ^24^ Department of Lower Gastrointestinal Surgery Kitasato University School of Medicine Kanagawa Japan; ^25^ Department of Surgery Kitasato University Kitasato Institute Hospital Tokyo Japan; ^26^ Department of Data Science National Cancer Centre Hospital East Chiba Japan

**Keywords:** ejaculatory dysfunction, rectal cancer, sexual function, ultra‐low rectal cancer

## Abstract

**Aim:**

The effect of laparoscopic surgery on sexual function in patients with ultra‐low rectal cancer remains unexplored. This multicentre study evaluated postoperative sexual function in male patients with rectal cancer located within 5 cm of the anal verge.

**Method:**

A total of 139 male patients aged ≤70 years with clinical T1‐2N0M0 rectal cancer underwent laparoscopic surgery between January 2014 and March 2017 at 47 institutions. Sexual function was assessed using the International Index of Erectile Function (IIEF‐15) and an ejaculation questionnaire preoperatively and at 3, 6, and 12 months postoperatively. Univariate and multivariate analyses were performed to examine risk factors for sexual dysfunction.

**Results:**

The IIEF‐15 scores showed a significant decrease at 3 months postoperatively, with partial recovery observed at 12 months; however, the scores remained below baseline levels. Age ≥ 56 years was identified as a significant risk factor for postoperative erectile dysfunction. Although ejaculatory function exhibited some improvement over 12 months, it did not return to preoperative levels. However, the orgasmic function, sexual desire, and overall satisfaction domains recovered close to their preoperative levels.

**Conclusion:**

Laparoscopic surgery for ultra‐low rectal cancer significantly affects male sexual function, particularly in older patients. These findings highlight the necessity for thorough preoperative counselling and targeted postoperative management to address sexual dysfunction.

**Originality Statement**

This study provides the first multicentre prospective analysis of postoperative sexual function following laparoscopic surgery for ultra‐low rectal cancer, identifying age as a key risk factor for erectile dysfunction and revealing limited recovery of ejaculatory function over 12 months.

## INTRODUCTION

Colorectal cancer is the fourth leading cause of cancer‐related deaths in men, accounting for 9.0% of all cancer‐related deaths [1]. Rectal cancer accounts for approximately 25% of all colorectal cancer cases [2]. The incidence of rectal cancer is projected to increase further in the coming years [3]. Laparoscopic surgery for rectal cancer has demonstrated long‐term efficacy [4], including through the use of robotic surgery [3, 4, 5] and transanal total mesorectal excision (taTME) [6, 7]. Furthermore, the effectiveness of the watch‐and‐wait approach after chemoradiotherapy has been documented, indicating the ongoing evolution of rectal cancer treatment [8, 9].

However, in addition to oncological outcomes, the postoperative health‐related quality of life must be maintained. Even with transanal total mesorectal excision (taTME) and autonomic nerve preservation for rectal cancer, sexual dysfunction is still reported to occur in 10%–59% of male patients [10, 11, 12, 13]. Studies comparing robotic, laparoscopic, and open surgeries have reported similar outcomes regarding sexual dysfunction [14, 15].

On the other hand, few studies have investigated erectile and ejaculatory function in patients with ultra‐low rectal cancer.

Recent reports indicate that patients desire preoperative information on sexual function, while surgeons tend to underestimate its importance [16]. Therefore, surgeons should pay attention to the postoperative sexual function in patients with rectal cancer.

The aim of our study was to evaluate postoperative sexual dysfunction in patients who underwent laparoscopic surgery for ultra‐low rectal cancer using the International Index of Erectile Dysfunction (IIEF‐15) questionnaire. Previous studies have reported a low response rate among female participants in surveys. However, given that this study focused specifically on ejaculatory and erectile function, we did not evaluate female sexual function [17]. Furthermore, the evaluation of female sexual function remains challenging due to the absence of a standardized methodological approach [18]. Consequently, this study predominantly focused on male participants.

## METHOD

This multicentre prospective study involved 47 institutions and 299 participants between January 2014 and March 2017 and was approved and overseen by the institutional review board of each participating hospital. The distribution of patient recruitment per institution was as follows: one institute recruited more than 50 patients, one recruited between 20 and 49 patients, six recruited between 10 and 19 patients, 11 recruited between five and nine patients, and 28 recruited between one and four patients (Table 1). The primary endpoint was assessed as sexual function.

**TABLE 1:** Proportion of registered cases based on institution.

Number of registered cases	Number of institutions
>50	1
49–20	1
19–10	6
9–5	11
4– 1	28

Written informed consent was obtained from all participants.

**FIGURE 1:**
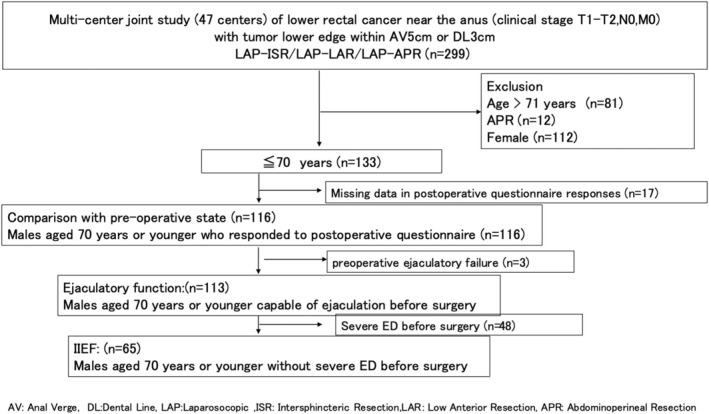
Patient selection flow chart. APR, abdominoperineal resection; AV, anal verge; DL, dental line; ISR, intersphincteric resection; LAP, laparosocopic; LAR, low anterior resection.

The inclusion criteria were as follows: clinical T1–T2, N0, and M0 (TNM classification), lower border of the tumour within 5 cm of the anal verge or 3 cm from the dentate line, laparoscopic intersphincteric resection or laparoscopic low anterior resection; and males aged 20 years or older diagnosed with adenocarcinoma. Exclusion criteria were as follows: clinical T3–4, *N* ≥ 1, M1 (TNM classification), females (*n* = 112), and severe preoperative erectile dysfunction was defined as a preoperative International Index of Erectile Function‐5 (IIEF‐5) score of 7 or lower (*n* = 41) (Figure 1).

The primary objective of this study was to evaluate the longitudinal recovery of postoperative sexual function. Accordingly, we included male patients aged between 20 and 70 years, as these individuals are more likely to retain or recover sexual function postoperatively. Furthermore, patients with severe preoperative erectile dysfunction (*n* = 48) or ejaculatory dysfunction (*n* = 3) were excluded, given that postoperative recovery was considered unlikely, thereby making it difficult to accurately assess longitudinal changes (Figure 1).

This study involved a subanalysis of the ULTIMATE trial data, with the number of analysed cases being selected based on the established inclusion and exclusion criteria (Figure 1).

### Quality control procedures

Only surgeons with an experience of more than 30 laparoscopic and 30 open operations for rectal carcinoma were approved for participation in this study by the study chair. We performed a central review of the surgical procedure based on examination of photographs obtained for all patients, one of which was a photograph of the pelvic floor after TME, and the other a photograph showing the level of mucosal incision via an anal procedure in cases for which ISR was performed.

### Evaluation method

The IIEF‐15 was used to assess sexual function. Questionnaires were administered preoperatively and 3, 6, and 12 months postoperatively. Erectile and ejaculatory functions were also evaluated. The follow‐up period was up to 1 year postoperatively.

As baseline measurements, we used data obtained in a preoperative survey conducted 2 weeks prior to surgery.

### Erectile function

The IIEF‐15 is a validated 15‐item self‐administered questionnaire for assessing erectile function, with a total score range from to 5–75 points [19]. The brevity and ease of comprehension provide practical advantages.

### Ejaculatory function

Questionnaires were administered preoperatively and 3, 6, and 12 months postoperatively. The items were: (1) able to ejaculate, (2) no ejaculation, (3) pain during ejaculation, and (4) orgasms without semen emission (retrograde ejaculation).

Patients diagnosed with sexual dysfunction were referred to the urology departments of the respective institutions for further consultation and treatment planning.

### Statistical analysis

Categorical variables are presented as counts (%) and continuous variables as medians and ranges. Longitudinal changes in the IIEF‐15 scores are shown as medians. Fisher's exact test and the Mann–Whitney U test was used to assess the differences between groups. The Wilcoxon signed‐rank test and Mann–Whitney U test was used to evaluate longitudinal changes in erectile function. Univariate and multivariate analyses were performed to examine risk factors for sexual dysfunction. The factors considered included surgical procedure (ISR vs. LAR), age (<56, ≥ 56), left colic artery (preservation or ligation), tumour location (anterior wall or other locations), operation time (< 300 or ≥ 300), and blood loss (35 < or ≥ 35). The cutoff value was established at 56 years, corresponding to the median age of the 65 cases included in the International Index of Erectile Function (IIEF) analysis. The median operative time was 282 min, and the median loss of blood was 34 mL; on the basis of these values, we set the cutoff for operative time at 300 min and blood loss at 35 mL.

Multivariate logistic regression was used to calculate odds ratios (ORs) and 95% confidence intervals (CIs). Ejaculatory function was independently analysed using Fisher's exact test. In this study, as opposed to using a complete case analytical approach, we applied a mixed‐effects model, which facilitates the analysis of data even in cases with missing values. JMP Pro 16 software was used for statistical analyses, with *p* < 0.05 considered statistically significant.

## RESULTS

### Patient characteristics

The median age was 61 years (range, 35–70 years). Clinical stage T1 cases accounted for 56.8% and clinical stage T2 for 43.1% of cases (Table 2). The median operation time was 282 (112–589) min with no conversion to open surgery. Anastomotic leakage occurred in 15 (10.8%) patients. The LCA was preserved in 62 patients (44.6%). Stomas were created in 127 (91%) patients. A total of 122 patients (87.7%) underwent stoma closure, with a median time from the initial surgery of 4 months (range, 1–33 months) (Table 3).

**TABLE 2:** Patient characteristics.

	*n* = 133
Age	60 (35–70)
Gender	
Male	133
Female	0
BMI	23 (16–34)
PS	
0	126 (94.7%)
1	7 (5.26%)
Tumour marker	
Carcinoembryonic antigen	2 (0.4–27.4)
Cancer antigen 19‐9	8 (0.1–71.2)
Clinical stage	
T1	76 (57.1%)
T2	57 (42.8%)
Comorbidity	
HT	43 (32.3%)
DM	19 (14.3%)
Tumour location	
Right lateral wall	20 (15.3%)
Posterior wall	46 (34.5%)
Left lateral wall	28 (21.1%)
Anterior wall	39 (29.3%)
Surgical procedure	
Hartmann	1 (0.7%)
LAR	51 (38.4%)
ISR	72 (54.1%)
CAA	9 (6.77%)

Abbreviations: APR, abdominoperineal resection; BMI, body mass index; CAA, coloanal anastomosis; DM, diabetes mellitus; HT, hypertension; ISR, intersphincteric resection; LAR, low anterior resection; PS, performance status.

**TABLE 3:** Surgical outcomes and pathological findings.

	*n* = 133
Operative times (min)	278 (113–589)
Bleeding (mL)	33 (0–450)
Conversion to laparotomy	0
Transfusion	0
Stoma creation	127 (95.4%)
Stoma closure	122 (96.1%)
Postoperative complications	
Anastomosis leakage	15 (11.3%)
Ileus	26 (19.5%)
Anastomosis stenosis	0
SSI	3 (2.25%)
LCA preservation	
Preservation	61 (45.8%)
Ligation	72 (54.1%)
Dissection	
D3	80 (60.2%)
D2	53 (39.8%)
Number of lymph nodes	10 (1–46)
Tumour size pathological T	
Is	2 (1.5%)
1	73 (54.9%)
2	45 (33.8%)
3	13 (9.77%)
4	0
Pathological N	
0	114 (85.7%)
1	16 (12.0%)
2	3 (2.26%)
Adjuvant chemotherapy	19 (14.3%)

Abbreviations: LCA, left colic artery; SSI, surgical site incision.

### Longitudinal changes in erectile function

Table 3 shows the trends of the mean IIEF‐15 and domain scores for all patients. The IIEF‐15 scores decreased at 3 months postoperatively, with slight improvement at 6 months and 1 year, but IIEF‐15 at 12 months postoperatively were significantly lower than those preoperatively (*p* < 0.001).

**FIGURE 2:**
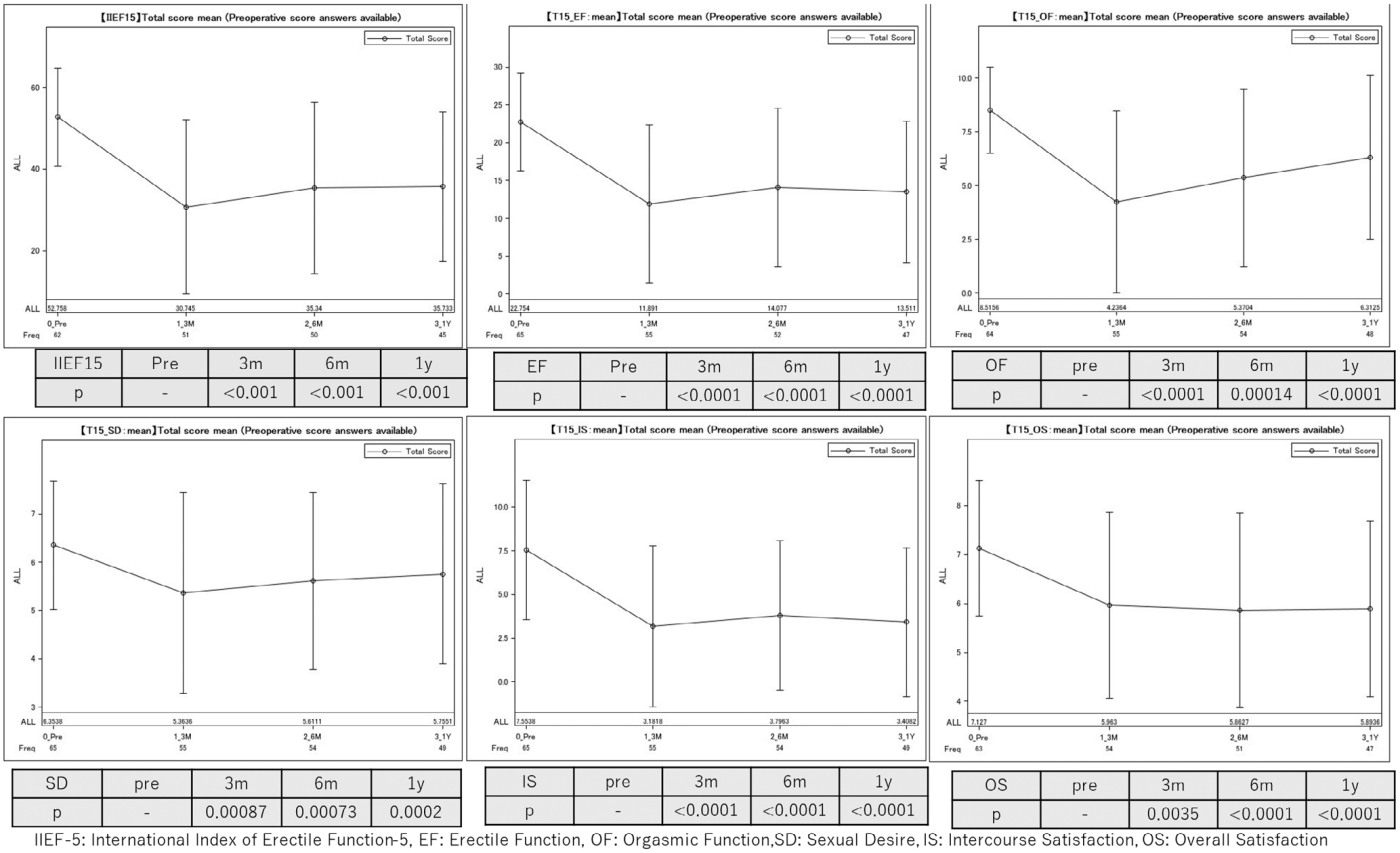
Longitudinal changes in erectile function (IIEF‐15). EF, erectile function; IIEF‐5, International Index of Erectile Function‐5; IS, intercourse satisfaction; OF, orgasmic function; OS, overall satisfaction; SD, sexual desire.

Changes in each domain of the IIEF‐15, specifically erectile function, sexual desire, intercourse satisfaction, orgasmic function, and overall satisfaction, demonstrated a decline at 3 months postoperatively. Although orgasmic function and sexual desire exhibited a gradual trend toward improvement at 6 months postoperatively, the scores for all domains remained significantly lower than the preoperative baseline values at 1 year postoperatively (*p* < 0.001) (Figure 2).

### Longitudinal analysis of temporal changes in ejaculatory function

**FIGURE 3:**
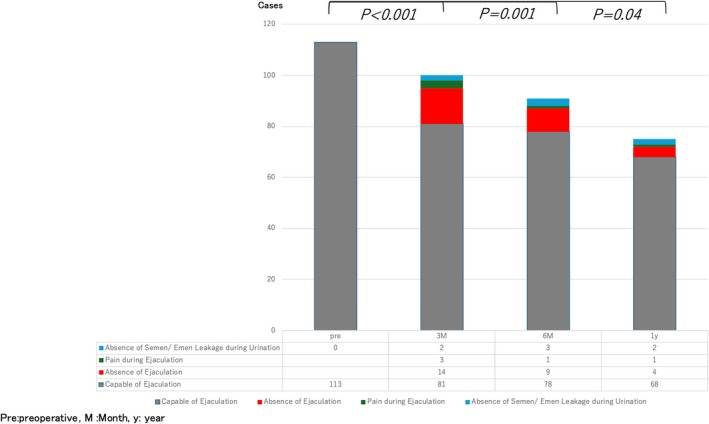
Longitudinal analysis of temporal changes in ejaculatory function. M, month; Pre, preoperative; y, year.

The proportion of patients unable to ejaculate was 14.4% at 3 months, 11.5% at 6 months, and 5.88% at 1 year postoperatively, showing a slight improvement from 3 months but remaining lower than preoperative levels (Pre vs. 3 M: *p* < 0.001; Pre vs. 6 M: *p* = 0.001; Pre vs. 1Y: *p* = 0.04) (Figure 3).

### Risk factors for erectile dysfunction

Multivariate analysis of risk factors including surgical procedure, age, LCA preservation, tumour location, operation time, blood loss, and stoma presence revealed age ≥ 56 years as a risk factor (*p* = 0.05).

### Risk factors for ejaculatory dysfunction

Multivariate analysis of factors, including surgical procedure, age, LCA preservation, tumour location, operation time, blood loss, and stoma presence, revealed no independent factors affecting ejaculatory function at 12 months postoperatively.

## DISCUSSION

This multicentre, prospective study evaluated postoperative sexual dysfunction in patients who underwent laparoscopic surgery for ultra‐low rectal cancer using the IIEF‐15 questionnaire. In our study, IIEF‐15 scores were lowest at 3 months postoperatively, with slight recovery at 6 and 12 months; however, scores remained significantly lower than the preoperative levels. Age ≥ 56 years was identified as a risk factor for erectile dysfunction. Regarding ejaculatory function, 14.4% of patients were unable to ejaculate at 3 months postoperatively, declining to 5.97% at 1 year; however, this did not improve to preoperative levels. No specific risk factors of ejaculatory dysfunction were identified.

Erectile dysfunction has been reported to occur due to arteriosclerosis, chronic ischaemia, and androgen deficiency associated with aging [20]. Furthermore, previous studies have also reported that age > 56 years is a risk factor for sexual dysfunction (IIEF‐5) [21], which is consistent with our finding that increasing age is a risk factor for erectile dysfunction [10, 22, 23]. Some studies comparing high and low ligation of the inferior mesenteric artery reported better IIEF‐5 scores with low ligation, possibly because of the proximity of the nerves in high ligation [24]. However, other studies found no difference between high and low ligation rates, which may be attributed to the precision of robotic surgery [25]. Our study found no association between ligation level and sexual function, possibly because of the maintained surgical quality across the 47 participating institutions.

Hanaoka et al. examined the risk factors for sexual dysfunction after robotic surgery for rectal cancer [25]. They identified chemoradiotherapy as a risk factor for erectile dysfunction, and chemoradiotherapy, lateral lymph node dissection, and postoperative adjuvant chemotherapy as risk factors for ejaculatory dysfunction. IIEF‐5 scores were lowest at 3 months postoperatively, with a recovery trend up to 1 year; however, this remained significantly lower than the preoperative levels. The orgasmic function, overall satisfaction, and sexual desire domains recovered to their preoperative levels at 12 months postoperatively. Wei et al. reported a recovery trend in both erectile and ejaculatory functions, although scores remained lower than preoperative levels at 12 months postoperatively [26]. While our study focused on clinical stage T1–2 cases, which differs from previous reports on patient backgrounds, our results were consistent with those of previous studies.

The inferior hypogastric plexus is a crucial branch of the pelvic autonomic nerves for ejaculatory function. Branches to the corpora cavernosa and seminal vesicles originate from the inferior hypogastric plexus, pass through the neurovascular bundles (NVBs) on both sides of the Denonvilliers' fascia (DVF), traverse the urogenital diaphragm to innervate the corpora cavernosa and seminal vesicles, and control erection and ejaculation. Han et al. reported that bilateral NVBs emit small communicating branches as they pass through the DVF, crossing the anterior surface of the DVF to connect the bilateral pelvic autonomic nerves [27]. Consequently, even if the bilateral NVBs are completely preserved during DVF incision, these small communicating branches are inevitably damaged, affecting postoperative sexual function. A meta‐analysis of 22 studies also reported that preservation of DVF influences the postoperative recovery of sexual function [28]. Although our study did not specifically examine the DVF, given that the target cases were cT1–2, it is likely that they were preserved, possibly explaining the gradual improvement in ejaculatory function over time. Furthermore, while 40% of the tumours were located on the anterior wall, tumour location was not identified as a risk factor for sexual dysfunction.

This study has several limitations. First, sexual dysfunction after rectal cancer surgery is multifactorial, and psychological factors such as libido, body image, and self‐esteem play important roles [29]. Additionally, the presence of stoma may have contributed to decreased sexual activity [30]. In our study, 91% of cases involved stoma creation, which may have influenced the results. Second, the evaluation of sexual function relied on questionnaires, which introduced a potential subjective bias. Third, this was a single‐arm study which lacked direct comparison with other surgical approaches, such as robotic surgery or taTME. Fourth, the study population was limited to male patients. Additionally, the study was restricted to T1–T2 tumours, with cases involving T3–T4 tumours or preoperative radiotherapy being excluded, so as to minimize the influence of surgical complexity and technical factors on outcomes. Furthermore, we did not assess data pertaining to urinary function. Finally, this study was conducted in specialized Japanese centres, necessitating caution when generalizing the results to other populations.

In conclusion, we evaluated sexual function after surgery in patients with cT1‐2, N0M0 lower rectal cancers. IIEF‐15 scores were the lowest at 3 months postoperatively, which gradually recovered; however, scores remained lower than the preoperative levels at 1 year. Ejaculatory function showed limited recovery at 12 months postoperatively. Given the implications of these results, it is imperative to ensure widespread awareness of erectile dysfunction risk among both surgeon and patient populations before surgery for rectal cancer.

## AUTHOR CONTRIBUTIONS

**Yasuhiro Ishiyama:** Conceptualization; methodology; software; investigation; validation; formal analysis; writing – review and editing; writing – original draft. **Yasumitsu Hirano:** Investigation; funding acquisition; visualization. **Yuichiro Tsukada:** Data curation; supervision; resources; project administration; investigation. **Jun Watanabe:** Investigation; funding acquisition; methodology; resources; supervision. **Yosuke Fukunaga:** Investigation; data curation; resources; supervision. **Kazuhiro Sakamoto:** Data curation; investigation; supervision. **Hiroki Hamamoto:** Investigation; data curation; resources; supervision. **Masanori Yoshimitsu:** Data curation; investigation; supervision. **Hisanaga Horie:** Investigation; data curation; supervision. **Nobuhisa Matsuhashi:** Resources; data curation; investigation; supervision. **Yoshiaki Kuriu:** Resources; data curation; formal analysis; supervision. **Shuntaro Nagai:** Resources; data curation; supervision. **Madoka Hamada:** Supervision; data curation; resources. **Shinichi Yoshioka:** Supervision; data curation; resources. **Shinobu Ohnuma:** Supervision; data curation; resources. **Tamuro Hayama:** Supervision; data curation; resources. **Koki Otsuka:** Supervision; resources; data curation. **Yusuke Inoue:** Supervision; data curation; resources. **Kazuki Ueda:** Data curation; supervision; resources. **Yuji Toiyama:** Data curation; supervision; resources. **Satoshi Maruyama:** Data curation; supervision; resources. **Shigeki Yamaguchi:** Supervision; data curation; resources. **Keitaro Tanaka:** Data curation; supervision; resources. **Takeshi Naitoh:** Data curation; supervision; resources. **Masahiko Watanabe:** Supervision; data curation; project administration; resources. **Motoko Suzuki:** Data curation; supervision; formal analysis; software; methodology; visualization; investigation. **Toshihiro Misumi:** Data curation; supervision; formal analysis; software; validation; visualization; investigation; conceptualization. **Masaaki Ito:** Conceptualization; investigation; funding acquisition; validation; methodology; software; formal analysis; data curation; supervision; resources; project administration; visualization.

## FUNDING INFORMATION

none.

## CONFLICT OF INTEREST STATEMENT

The authors have no conflicts of interest directly relevant to the content of this article. The authors declare that no funding was received in support of this study.

## ETHICS STATEMENT

The study design was approved by the appropriate ethics review board.

## TRIAL REGISTRATION

UMIN Clinical Trials Registry number, UMIN000011750.

## Data Availability

The data that support the findings of this study are available from the corresponding author upon reasonable request.
